# CopA3 Peptide Prevents Ultraviolet-Induced Inhibition of Type-I Procollagen and Induction of Matrix Metalloproteinase-1 in Human Skin Fibroblasts

**DOI:** 10.3390/molecules19056407

**Published:** 2014-05-20

**Authors:** Dong-Hee Kim, Han-Hyuk Kim, Hyeon-Jeong Kim, Hyun-Gug Jung, Jae-Myo Yu, Eun-Su Lee, Yong-Hun Cho, Dong-In Kim, Bong-Jeun An

**Affiliations:** 1 Korea Promotion Institute for Traditional Medicine Industry, Gyeongsan 712-260, Korea; 2 Advanced Medical Fusion Textile Center, Gyeongbuk Technopark Foundation, Gyeongsan 712-210, Korea; 3 Department of Cosmeceutical Science, Daegu Haany University, Gyeongsan 712-715, Korea; 4 Radiation Division for Biotechnology, Advanced Radiation Technology Institute, Jeongeup Campus of Atomic Energy Research Institute (KAERI), Jeonbuk 580-185, Korea

**Keywords:** *Copris tripartitus*, CopA3, ultraviolet, matrix metalloproteinase-1, CCD-986sk cells

## Abstract

Ultraviolet (UV) exposure is well-known to induce premature aging, which is mediated by matrix metalloproteinase-1 (MMP-1) activity. A 9-mer peptide, CopA3 (CopA3) was synthesized from a natural peptide, coprisin, which is isolated from the dung beetle *Copris tripartitus*. As part of our continuing search for novel bioactive natural products, CopA3 was investigated for its *in vitro* anti-skin photoaging activity. UV-induced inhibition of type-I procollagen and induction of MMP-1 were partially prevented in human skin fibroblasts by CopA3 peptide in a dose-dependent manner. At a concentration of 25 μM, CopA3 nearly completely inhibited MMP-1 expression. These results suggest that CopA3, an insect peptide, is a potential candidate for the prevention and treatment of skin aging.

## 1. Introduction

When skin is frequently exposed to ultraviolet (UV) irradiation, which leads to photoaging. The photoaging process results in major skin alterations through stimulation of multiple signal transduction pathways, which lead to activation of transcription factors or target genes [1]. Many proteases are induced by UV and matrix metalloproteinases (MMPs) are especially critical enzymes responsible for UV-induced skin aging. The extracellular matrix (ECM) is important for skin structure and elasticity. About 80%–85% of ECM is consisted of type-I procollagen [2]. Procollagen was secreted and N- and C-terminal ends were truncated to form mature collagen [3]. Until now, 28 MMP genes have been discovered, including MMP-1 (interstitial collagenase) [4]. When skin was induced to UV, MMP-1 expression was increased, which broken down collagens and caused skin aging [5]. Therefore, we also tried to focus on MMP-1 which is relatively well known aging related gene expressions.

Coprisin is a natural peptide consisting of 43 amino acids made by the *Copris tripartitus*, a Korean dung beetle [6]. The isolation and characterization of a defensin-like peptide, coprisin, from the bacteria-immunized dung beetle was previously reported [7]. It was reported that the activity domain of the coprisin was the α-helical segment, peptide (LLCIALRKK) corresponding to this domain that was produced. This peptide, termed “CopA3”, was dimerized through a disulfide bonding, which occurs by cysteine in the middle position of the peptide [8]. Previous study has reported that the D-type, protease-resistant form of the CopA3 peptide has antimicrobial activity against *Clostridium difficile*, which causes acute gut inflammation in humans and animals [8]. In addition, the CopA3 inhibited lipopolysaccharide-induced macrophage activation [9]. After UVB exposure, skin fibroblasts are thought to produce oxidative free radicals [10] and undergo an inflammatory process [11]. A recent study has demonstrated that retinoic acid prevents photoaging from UV irradiation by altering multiple signal transduction pathways [1]. Taken together, this evidence suggests that CopA3 may act as a potent inhibitor of MMP-1 when fibroblasts were affected by UVB irradiation, so in this study, we investigated the effect of the insect-derived peptide CopA3 on the expression of MMP-1 and type-I procollagen in UVB-induced CCD-986sk cells.

## 2. Results and Discussion

### 2.1. CCD-986sk Fibroblast Cell Viability Measured by MTT Assay

**Figure 1:**
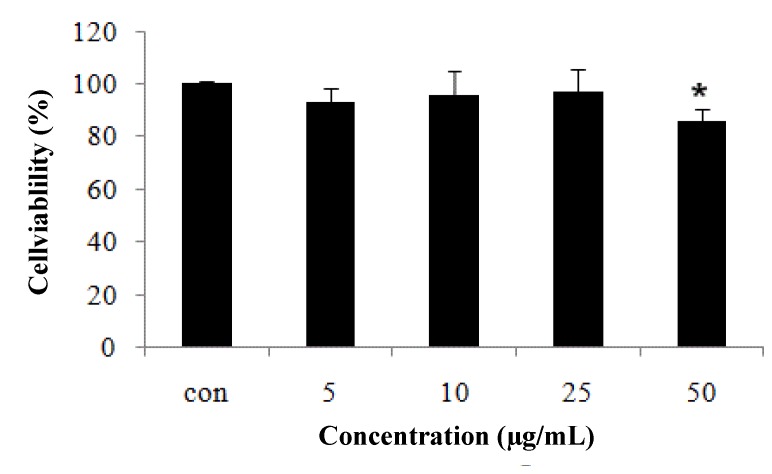
Cell viability of fibroblast CCD-986sk cells after CopA3 treatment. Fibroblast cells were exposed to UVB for 1 min and treated with various concentrations of CopA3. Cell viability was measured by MTT assay. All experiments were performed in triplicate. Values shown are means ± SEM (*n* = 3). * p < 0.05 *versus* the control group.

In preliminary experiments, the toxicity of various concentrations of CopA3 (5, 10, 25, and 50 µg/mL) toward CCD-986sk cells was tested. Cell viability was recalculated from cell toxicity. Cell viability was reduced markedly by 50 µg/mL, but viability loss was minimal up to 25 µg/mL (Figure 1). Therefore, 5, 10, and 25 µg/mL samples were used for further experiments. 

### 2.2. Effect of CopA3 on Type-I Procollagen Synthesis and Matrix Metalloproteinase-1 Levels

**Figure 2:**
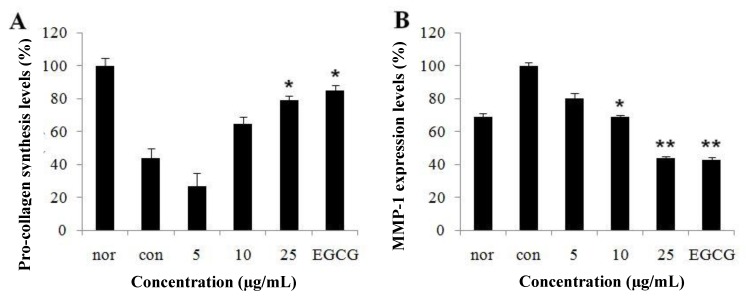
(**A**) Type-I procollagen synthesis and (**B**) MMP-1 inhibition of fibroblast CCD-986sk cells after CopA3 treatment. Fibroblast cells were exposed to UVB for 1 min and treated with various concentrations of CopA3. Type-I procollagen synthesis and MMP-1 inhibition were measured with an assay kit. All experiments were performed in triplicate. Values shown are means ± SEM (*n* = 3). * p < 0.05; ** p < 0.01 *versus* the control group. EGCG was used at 25 µg/mL.

UVB-induced human fibroblasts would stimulate the expression of genes MMP-1. We explored whether UVB-induced MMP-1 production was responsible for collagen destruction and photoaging and whether CopA3 had an ability to inhibit secretion of its enzymes in UVB-induced human fibroblasts. The UVB irradiation group showed more decreased type-I procollagen levels than the non-UVB irradiated group. This shows the decreased the effect of UVB irradiation on type-I procollagen content in CCD-986sk. CopA3 treatment increased UVB-induced type-I procollagen expression levels in a dose-dependent manner in the CCD-986sk cell line (Figure 2A). MMP-1 levels were also more enhanced by exposure of skin fibroblasts to UVB irradiation group at 20 mJ than in the non-UVB irradiated group. Treatment of CCD-986sk with CopA3 at 5, 10 and 25 µg/mL caused a decrease in these levels by about 60% (Figure 2B). These results demonstrate that CopA3 peptide increased the production of type-I procollagen and decreased the production of MMP-1 in UVB-induced fibroblast cells, suggesting that CopA3 may stimulate expression of type-I procollagen and/or inhibit MMP-1 gene expression in UVB-induced fibroblast cells.

### 2.3. mRNA of MMP-1 was Decreased in CCD-986sk Cells by CopA3

**Figure 3:**
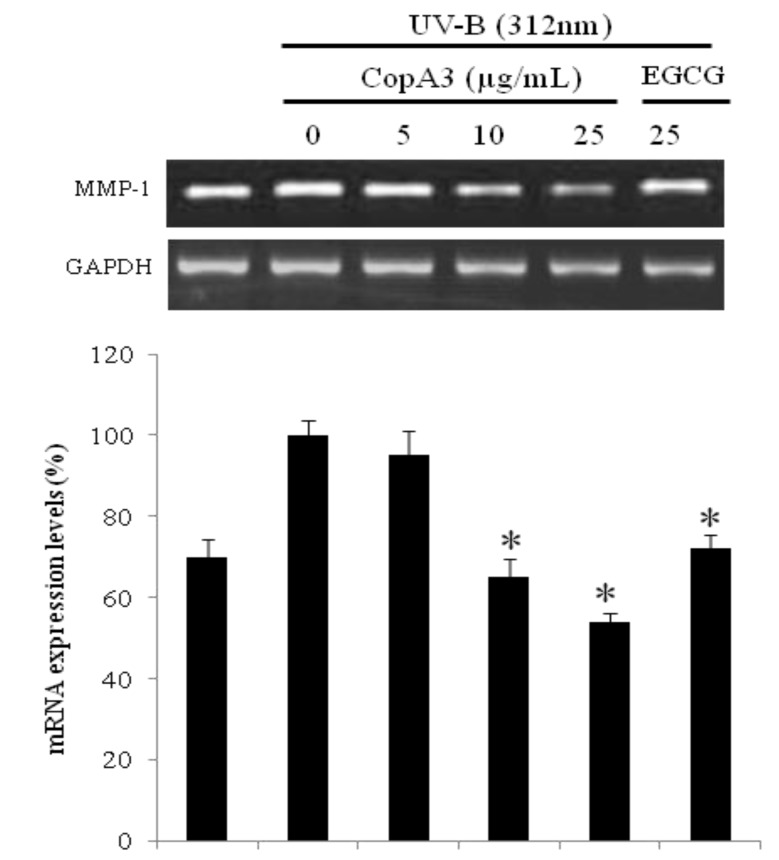
The mRNA expression of MMP-1 was evaluated by RT-PCR after CopA3 treatment on fibroblast CCD-986sk. After CCD-986sk cells (5 × 10^5^ cells) were grown and exposed to UVB for 1 min, the cells were treated with 5, 10 and 25 µg/mL of CopA3*.* The mRNA transcripts were detected by RT-PCR. GAPDH was used as internal control. All experiments were performed in triplicate. Values shown are means ± SEM (*n* = 3). * p < 0.05 *versus* the control group.

UVB induction of untreated CCD-986sk cells up-regulated the mRNA expression of MMP-1; however, CopA3 treatment suppressed the UVB-induced up-regulation of MMP-1 (Figure 3). CopA3 at a concentration of 25 µg/mL significantly decreased the expression of MMP-1 to basal levels. Treatment with 25 µg/mL CopA3 resulted in a 2-fold decrease in MMP-1 expression relative to untreated UVB-induced cells. The MMP-1-suppressing effect of CopA3 treatment was greater than those of epigallocatechin 3-O-gallate (EGCG), which is known to be a natural anti-aging agent. These results suggest that CopA3 is a potential candidate for the prevention and treatment of skin aging.

### 2.4. UVB Induced Aging Related MMP-1 Protein Expression was Suppressed by CopA3

**Figure 4:**
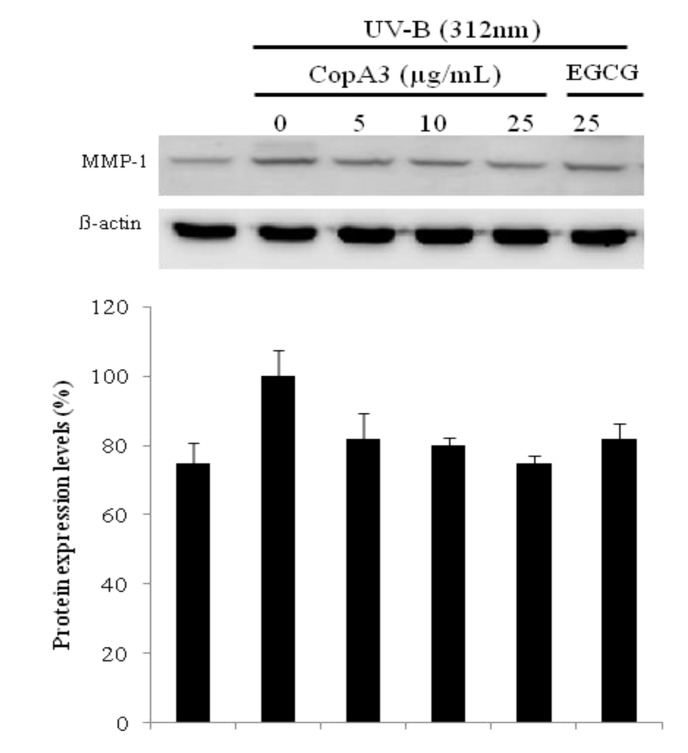
Protein expression of MMP-1 was compared after treatment of CopA3 on fibroblast cell CCD-986sk by western blotting. After CCD-986sk cells (5 × 10^5^ cells) were grown and exposed to UVB for 1 min, the cells were treated with 5, 10 and 25 µg/mL of CopA3*.* Protein expression was evaluated by western blotting. Antigen specific antibodies as primary antibodies and horse radish peroxidase (HRP) conjugated secondary antibody were used. Chemiluminescence was detected by ECL. All experiments were performed in triplicate. Values shown are means ± SEM (*n* = 3).

MMP-1 protein expression profiles were monitored by western blotting (Figure 4). After exposure to 20 mJ/cm^2^ UV induction, fibroblasts were incubated for an additional 48 h in the presence (or absence) of CopA3 (5–25 μg/mL). Treatment with CopA3 inhibited UV-induced MMP-1 expression by 19% at 5 μg/mL, 20% at 10 μg/mL and 22% at 25 μg/mL. The reduced protein levels of MMP-1 correlated well with the levels of MMP-1 mRNA expression. Natural products from medicinal plants are potential sources of MMP-1 inhibitors. Recently, several MMP-1 inhibitors from natural peptides have been identified, such as prepro-human urotensin II [12], and chlorella-derived peptide [13], but there has been no previous report of an insect-derived peptide with MMP-1 inhibitory activity to our knowledge. In the present study, we found that CopA3 prevented the UV–induced decrease in type-I procollagen expression and increment of MMP-1 expression. These results suggest that the protective effects of CopA3 on UVB-induced decrease in type-I procollagen and MMP-1 expression.

## 3. Experimental

### 3.1. CopA3 (Disulfide Dimer) Synthesis, Dimer Peptide Structure Determination

The insect-derived coprisin peptide CopA3 was synthesized by AnyGen (Gwang-ju, South Korea) [8]. The peptide was purified by reverse-phase high-performance liquid chromatography (HPLC) using a Capcell Pak C_18_ column (Shiseido, Tokyo, Japan); a linear gradient of water–acetonitrile (0%–80%) containing 0.1% trifluoroacetic acid was used to elute the peptide (45% recovery). The identity of the peptide was confirmed by ESI mass spectrometry (Platform II; Micromass, Manchester, UK). The interchain disulfide bond was formed by dissolving the synthetic peptide in an acetonitrile/H_2_O (50:50) solution and then oxidized by incubating in an aqueous 0.1 M NK_4_HCO_3_ solution (pH 6.0–6.5) for 24 h. The disulfide pattern of the dimeric form of CopA3 was determined by analyzing the peptide solution by HPLC and ESI mass spectrometry.

### 3.2. Cell Culture

CCD-986sk human fibroblast cell lines were purchased from ATCC (Manassas, VA, USA). All media required for cell growth were purchased from Gibco BRL (Franklin Lakes, NJ, USA). Cells were grown in 10% FBS and DMEM with 1% penicillin/streptomycin (100 U/mL) at 37 °C in a 5% CO_2_ incubator. Cells were stained with trypan blue and assessed for dye exclusion to eliminate dead cells, and then counted using a hemocytometer. Aliquots (100 μL) of cell suspension were plated in 96-well plates to 5 × 10^4^ cells/well and exposed to UVB at 312 nm (20 mJ/cm^2^) for 1 min (Bio-Sun lamps, Vilber Lourmat, Marne, France). A total of 20 μL of each sample concentration was added to cells while the same volume of water was added to the control. Preparations were grown for 48 h and used for further experiments.

### 3.3. Cell Viability

3-[4,5-Dimethylthiazol-2yl]-2,5-diphenyl-tetrazoliumbromide (MTT) was purchased from Sigma-Aldrich (St. Louis, MO, USA). The MTT assay was performed by a modification of a previously published protocol [14]. A total of 20 μL of MTT solution (5 mg/mL) was added and incubated for 4 h. After the MTT solution was removed, 150 μL dimethylsulfoxide was added and incubated for 30 min. The absorbance of each well was read for cell toxicity at 540 nm using an ELISA plate reader. 

### 3.4. Determination of MMP-1 and Type-I Procollagen

To measure MMP-1 (Abcam, Cambridge, MA, USA) and type-I procollagen (Takara, Shiga, Japan), the CCD-986sk cells were plated in 24-well plates (1 × 10^5^ cells/well). After 48 h, cells were incubated in the absence or presence of sample for additional time periods. At the end of treatment, cell culture supernatants were collected and analyzed for MMP-1 and type-I procollagen contents by ELISA according to the manufacturer’s protocol. Total RNA was extracted by Trizol (Invitrogen, Carlsbad, CA, USA) followed by manufacture’s protocol.

### 3.5. cDNA Synthesis and RT-PCR

Concentration of total RNA was measured by spectrophotometer. Two µg of RNA were used for cDNA synthesis with oligo (dT) primer and reverse transcriptase (Promega, Madison, WI, USA) for 1 h at 42 °C. PCR was performed with cDNA to analyze transcript expression of MMP-1 with 55 °C annealing temperature for 30 sec, 72 °C, 1 min for extension with total of 30 cycles. Transcripts were separated in 1.5% agarose gel and stained with ethidium bromide (EtBr) and confirmed by LAS4000 image analyzer (Fujifilm Life Science, Tokyo, Japan). The levels of transcripts were divided by the level of an internal control by Image J software. Primer sequences are listed in Table 1.

**Table 1:** Sequence information of primers used for RT-PCR.

Gene	Primer	Sequence (5' → 3')
MMP-1	Forward	AGC GTG TGA CAG TAA GCT AA
	Reverse	GTT TTC CTC AGA AAG AGC AGC AT
β-actin	Forward	ATT GTT GCC ATC AAT GAC CC
	Reverse	AGT AGA GGC AGG GAT GAT

### 3.6. Western Blotting

For western blot analysis with whole cell lysates, CCD-986sk cells were harvested by centrifugation at 4 °C 12,000 rpm for 20 min after adding 10 mL of radio immunoprecipitation assay (RIPA) buffer with protease inhibitor cocktails. Supernatant was saved, and protein concentration was measured by Bradford assay [14]. A total of 20 µg of each protein were separated by 10% of SDS-PAGE and transferred onto a PVDF membrane by tank transfer. Primary antibody was added to 1:1,000 and stored at 4 °C overnight. The membrane was washed with TBST for 3 times, either mouse anti-rabbit IgG-HRP for MMP-1 was added as secondary antibody (1:1,000) and incubated for 2 h at RT. After 3 times TBST washing, Chemiluminewcence was detected by SuperSignal West Pico chemiluminescent substrate (Pierce, Rockford, IL, USA). The protein signal was analyzed by LAS 4000 image analyzer (Fuji Film Life Science, Tokyo, Japan).

## 4. Conclusions

In summary, the data indicate that CopA3 peptide is a potent anti-photodamaging constituent of *Copris tripartitus*. CopA3 peptide decreased the production of MMP-1, and increased the production of type-I procollagen in UVB-induced human fibroblast. These results imply that CopA3 peptide might be beneficial for the treatment of skin aging.
